# Regulation of asymmetric cell division in the epidermis

**DOI:** 10.1186/1747-1028-6-12

**Published:** 2011-06-06

**Authors:** Samriddha Ray, Terry Lechler

**Affiliations:** 1 Department of Cell Biology, Duke University Medical Center, Durham, USA

## Abstract

For proper tissue morphogenesis, cell divisions and cell fate decisions must be tightly and coordinately regulated. One elegant way to accomplish this is to couple them with asymmetric cell divisions. Progenitor cells in the developing epidermis undergo both symmetric and asymmetric cell divisions to balance surface area growth with the generation of differentiated cell layers. Here we review the molecular machinery implicated in controlling asymmetric cell division. In addition, we discuss the ability of epidermal progenitors to choose between symmetric and asymmetric divisions and the key regulatory points that control this decision.

## Introduction

Asymmetric cell divisions (ACDs) generate cellular diversity during the development of multi-cellular organisms from a single-celled embryo. The asymmetric division of a progenitor cell generates two daughters with non-identical cell fates, typically one daughter remains a progenitor while the other commits to a defined cell lineage through differentiation [1]. During development and in adult stem cells, ACDs allow for the maintenance of the stem/progenitor cell pool as well as the generation of differentiated cells. While adult stem cells can also undergo symmetric divisions with subsequent differentiation, there is now compelling data that a number of tissue-specific stem/progenitor cells, including those of the neuronal, hematopoietic, muscle and epidermal lineages, undergo ACD [2-6]. This work was made possible and heavily influenced by pioneering studies on ACD in *D. melanogaster *and *C. elegans*, which has been reviewed in detail elsewhere [7,8]. Recent work has highlighted the advantages of studying ACD in the epidermis, with novel advances in understanding the many levels at which this process is regulated [5,9,10]. One crucial determinant of ACD is the axis of spindle orientation and it regulation, which is the major focus of this review.

## Discussion

### Epidermal Development

**Figure 1:**
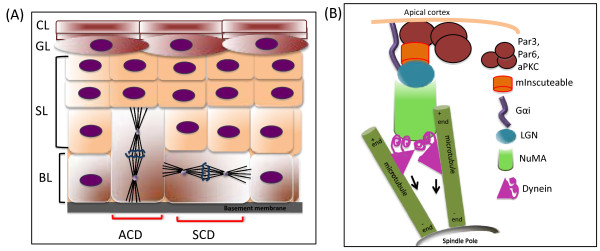
**Asymmetric division of epidermal progenitor cells through perpendicular spindle orientation promotes stratification**. (A) Epidermis is a multi-layered structure whose inner most layer, the basal layer (BL), contains progenitor cells that can orient their mitotic spindle parallel or perpendicular to the underlying basement membrane (BM) to undergo symmetric (SCD) or asymmetric division (ACD) respectively. Asymmetric division of a basal cell gives rise to a differentiated suprabasal/spinous layer (SL) cell and a proliferative basal cell. Cells of the SL further differentiate and migrate outward to give rise to the granular layer (GL) and the cornified layer (CL). (B) Coupling between the apical Par polarity complex and NuMA in mitosis is proposed to direct apical-basal spindle orientation to drive ACD in the epidermal basal cells.

The epidermis is a multi-layered epithelium. Development of the epidermis from a single layer of epithelial progenitors is termed stratification (Figure 1A). The innermost cell layer, called the basal layer, lies on top of a basement membrane separating it from the underlying dermis. Cells of this layer undergo symmetric divisions to increase surface area through much of development. Starting around embryonic day 13.5 in the mouse, division orientation changes and the majority of divisions occur with the mitotic spindle oriented along the apical-basal axis of the cell [5,11]. These divisions are referred to as asymmetric. Definitive evidence for cell fate asymmetry of these divisions was recently reported [9]. Short-term lineage tracing of dividing progenitor cells revealed that those with spindles perpendicular to the basement membrane gave rise to one basal cell that expressed keratin-14 and one suprabasal cell that expressed keratin 10 [9]. Therefore, there is a clear and direct correlation between spindle orientation and cell fate decisions in the epidermis.

While the connection between spindle orientation and cell fate is incompletely understood, there is evidence for both intrinsic and extrinsic regulation. The basement membrane is a "niche" for progenitors and displacement from that niche, as happens during ACD, is likely to play a significant role in cell fate decisions. The integrin family of proteins mediates attachment of the basement membrane to the underlying extracellular matrix (ECM) and integrin-ECM signaling has been implicated in the maintenance of the proliferative state of epidermal basal cells [12,13]. Whether physical detachment from the BM is a necessity to trigger the switch from proliferative to differentiated state needs further investigation. In addition, both EGFR and Notch signaling have been implicated as intrinsic factors that may control the cell fate decision [10,14,15].

### Basal Progenitors Have a Choice in Division Orientation

From the onset of stratification until birth, epidermal progenitors balance ACD and SCD (symmetric cell division) to provide a tissue of the right size and thickness. To accomplish this, two extreme models for progenitor organization have been proposed. In one, all cells are committed to either ACD or SCDs. In this case, by balancing the relative proliferation rates of the two types of cells, a tissue could achieve proper development. Alternatively, every cell could have the ability to divide in each orientation. In this case the collective choices that individual cells make would provide the balance. This would suggest that each cell must survey its environment and make a decision each time it divides - asymmetric or symmetric [16,17]. Of course, a combination of these could also occur (i.e. a small population of cells committed to a division orientation with others choosing). While an ideal answer to this question would come by imaging repeated cell division cycles in an intact embryo, this has not yet been possible. Instead short-term lineage tracing studies have been performed which allow the clonal analysis of cells over two divisions cycles where their progeny can be easily classified. Analysis of the results indicated that the majority of basal cells are able to divide both symmetrically and asymmetrically [9].

How does a basal cell regulate its mode of division? As mentioned earlier an essential determinant of asymmetric division is the orientation of the mitotic spindle. Therefore understanding both the machinery that drives spindle reorientation as well as its regulation is paramount.

### Molecular Mechanisms Underlying Spindle Reorientation

Spindle reorientation has been extensively studied in a number of invertebrate model systems and is especially well understood during fly nervous system development [7,8,18]. Mechanistic studies of the process in *Drosophila *neuroblasts have unveiled the existence of a close interplay between polarity complex and microtubule-associated spindle regulators in regulating the apicobasal spindle orientation. Neuroblast apical-basal polarity is defined by the asymmetric distribution of the Par complex [19,20]. Apically localized Par complex, comprised of Par3-Par6-aPKC (atypical protein kinase), serves as a cortical marker for the apico-basal orientation of the mitotic spindle in these cells. Par3 binds and recruits the adaptor proteins Inscuteable and Pins (Partner of Inscuteable) to the apical cortex [19,20]. Recruitment of Pins to the apical cortex is further facilitated by its interaction with the Gαi subunit of the heterotrimeric G protein complex via its GoLoco motif [21,22]. Cortical Pins in turn recruits the *Drosophila *homolog of mammalian NuMA called MUD [23-25]. Based on its similarity to NuMA and LIN-5 (*C.elegans*), it is likely that MUD associates with microtubules and the minus end directed motor protein, dynein. Thus apically bound NuMA/MUD is hypothesized to act as a cortical anchor for dynein and microtubules which generate a pulling force on the astral microtubules leading to apical-basal spindle orientation [18,26]. Similar to the neuroblasts, epidermal progenitor cells exhibit a coupling between the polarity complex and spindle machinery to drive perpendicular spindle orientation in the cells undergoing ACD [5]. Loss of function analysis of LGN and NuMA in epidermal progenitors has demonstrated conserved functions in spindle orientation, failure of which results in impaired differentiation and architecture of the epidermis [8].

Interestingly, in neuroblasts a second spindle orientation pathway has been uncovered that involves interaction of Pins with the tumor suppressor Discs large (Dlg) and microtubule plus-end-directed kinesin heavy chain 73 (khc73) in regulation of ACD [27,28]. It remains unknown if epidermal progenitors also have back-up mechanisms to promote ACD and will require further investigation.

### Temporal Regulation of Spindle Orientation

Because basal cells can divide symmetrically or asymmetrically, this necessitates a choice of division orientation during each mitosis. As a population, the epidermal progenitors must balance SCD and ACD to allow for proper surface area growth at the same time that stratification is occurring. While *Drosophila *neuroblasts have served as an invaluable model for discovering the molecular machinery involved in ACD, they do not offer insight into this problem. This is because neuroblasts obligately divide asymmetrically - thus the cells do not need to integrate extrinsic signals to decide upon a division orientation. The epidermis, in contrast, offers an ideal system to understand how progenitor cells make this choice. Importantly, asymmetric division is predicted by the spindle orientation, thus allowing an easily visualized readout of this decision. When do cells choose division orientation? Careful analysis in embryos demonstrated that spindle orientation remained random in early mitosis and was stabilized only by late metaphase [9]. This is in contrast to embryonic and larval neuroblasts which pre-pattern their spindle axis even before nuclear envelope breakdown [29]. Because the neuroblasts always divide asymmetrically, this early establishment is a viable option. For epidermal progenitors, the late establishment of spindle orientation by rotation during metaphase may allow them to respond acutely to environmental changes. It is surprising that the very similar machinery in epidermal progenitors and *Drosophila *neuroblasts achieve the same final result (spindle orientation) through somewhat distinct mechanisms. These data suggest a level of regulation by cell cycle machinery that may include both localization and activation of the ACD apparatus. For example, in *Drosophila *the NuMA homolog, Mud, is not trapped in the nucleus and therefore does not rely on nuclear envelope breakdown to localize to the apical cell cortex. This may allow its earlier localization and the earlier capture of one of the centrosomes/spindle poles. However, regulation of the activity of the ACD machinery may also occur during mitosis. In *C. elegans *embryonic development, the timing of spindle forces and displacement are cell-cycle regulated [30]. Similarly, rotation of the mitotic spindle in epidermal progenitors appears to be activated at an ill defined time point. This suggests that the machinery waits, poised for activation. Additional roles for cell cycle machinery in regulating ACD comes from the observation that not only are the levels of mInscuteable and LGN cell-cycle regulated, but so is their localization to the apical cortex [5,9]. Whether this is due to modifications that directly affect Par3/mInscuteable interactions is currently under investigation. In *Drosophila *neuroblasts it is clear that cell cycle regulators, such as Aurora A, act through the polarity complex and aPKC to control the localization of cell fate determinants, though roles in spindle orientation have not been described [31]. Whether this pathway operates in the epidermis has not been addressed.

A fulfilling answer to the question of when a cell decides to divide asymmetrically has not yet been achieved. Based on the spindle orienation studies and expression patterns of ACD machinery [5,9], it is reasonable to speculate that transcriptional control is a key regulatory point during epidermal development.

### mInscuteable Expression is Sufficient to Drive ACD

Extensive research towards understanding the molecular mechanisms of spindle orientation and cell fate determination in *Drosophila *CNS development led to the identification of Inscuteable as a critical regulator of neuroblast asymmetric division [32]. Inscuteable is specifically expressed in and required for ACD of the neuroblasts but is absent from the symmetrically dividing neuroectoderm. Ectopic expression of the protein in neuroectoderm induced spindle reorientation, similar to what is seen in neuroblasts [32,33]. These observations demonstrate both necessity and sufficiency of Inscuteable in ACD. However, if mInscuteable (mInsc) has a similar role in spindle orientation and ACD of epidermal basal cells remained unclear. mInsc is an ideal candidate to control this decision, as it shows a restricted expression pattern and because it links the cortical polarity complex with the mitotic machinery.

To assess whether mInsc was sufficient for inducing spindle reorientation, mice that allowed epidermal-specific and doxycycline-regulated mInscuteable expression were generated. This allowed high spatial and temporal control of expression. Short-term (8 hour) induction of mInscuteable expression in the epidermis substantially enhanced the population of asymmetrically dividing cells. This underscored the critical role of mInscuteable in inducing perpendicular spindle orientation in the epidermis. Consistent with previous results, ectopically expressed mInsc maintained its apical localization and co-localized with LGN and the microtubule binding protein NuMA [9]. These observations suggested that mInsc can drive apical localization of other known components of the ACD machinery making it an upstream regulator of epidermal stratification. Importantly, this also increased the fraction of asymmetric cell divisions as determined by a lineage-tracing approach [9].

While mInsc is sufficient to induce ACD, it remains unknown whether it is required for them. Loss of function studies are required to determine whether it is necessary or whether there are redundant mechanisms for either spindle orientation and/or stratification. In further support of mInsc's role in driving spindle reorientation, it undergoes pronounced developmental up regulation at the onset of stratification [9]. Therefore, elucidating the developmental control of mInsc transcription will be essential in understanding stratification.

### Robustness in Asymmetric Divisions in the Epidermis

Although mInsc over-expression generated a marked shift towards asymmetrically dividing cells in short-term inductions, this was not maintained over longer time points. Unexpectedly, prolonged expression of mInsc resulted in a decrease in the percentage of asymmetrically dividing cells [9]. Therefore, the tissue was able to respond to an imbalance in cell division ratios and correct it.

The mechanism underlying this robustness has begun to be understood. It is not due to loss of expression or apical localization of mInsc. Similarly, LGN is still recruited to the apical cell cortex where it co-localizes with mInsc. In these cases a cortical crescent of mInsc/LGN is clearly uncoupled from the orientation of the mitotic spindle. These results are intriguing because a number of recent papers have suggested that LGN is required for symmetric cell divisions in simple epithelia [34-36]. This data and additional loss of function data argue that LGN is not required for symmetric cell divisions in the epidermis [10]. Therefore, while great progress has been made in identifying the molecular machinery required for ACD in the epidermis, we still know very little about SCD.

**Figure 2:**
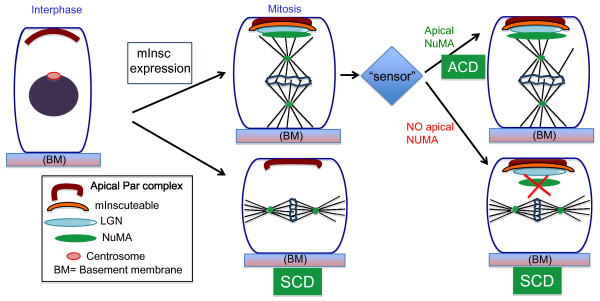
**Robust control of perpendicular spindle orientation in epidermal basal cells**. Wild-type cells expressing intrinsic ACD factors like mInsc localize mInsc/LGN/NuMA at the apical cortex in early mitosis but do not establish stable perpendicular spindle until late metaphase. As described in text, recent results have showed that spindle orientation can be regulated downstream of Insc/LGN recruitment by regulating NuMA maintenance at the apical end. Thus cells have mechanisms for robust regulation of mitotic spindle orientation to ensure optimum balance between SCD and ACD. Such mechanisms might help to respond quickly to extrinsic cues or intrinsic imbalances.

While mInsc/LGN localization are normal after prolonged mInsc expression, the localization of NuMA is notably altered. In all mitotic cells NuMA localizes to the spindle poles. It also localizes to the apical cell cortex (where it colocalizes with mInsc) in asymmetric divisions. This cortical localization was lost after prolonged mInsc expression. Thus the mechanism for robustness of spindle orientation relies on regulated recruitment of NuMA to the apical cell cortex. Evidence that this level of regulation occurs physiologically came from analysis of wild-type embryos. While there is a strong correlation between apical mInsc/LGN and spindle orientation, it is not absolute (i.e. they are uncoupled in wild-type embryos at a low rate). Together these data suggest that mInsc levels dictate a baseline for ACD rate, but that this can be acutely overcome during mitosis by regulating NuMA recruitment (Figure 2). This would allow a cell to quickly respond to extrinsic cues.

### Function of p63 in ACD

p63 is often viewed as a master regulator of stratification due to the profound defects caused by its loss. The ectoderm cells do not commit fully to epidermis and no stratification/differentiation occur [37-40]. p63 was therefore a very good candidate for a transcriptional regulator of mInsc expression. However, examination of mInsc levels in p63 null embryos demonstrated normal expression [9]. Additionally, while late-stage embryos undergo predominantly symmetric cell divisions, embryos at earlier stages exhibit randomized division orientations. The late phenotypes are likely due to decreased proliferation in the epidermal progenitors. As the embryo rapidly grows, the surface ectodermal cells become stretched over the surface and are forced into symmetrical divisions.

If mInsc is expressed, why do these cells not undergo ACD and stratify? Examination of protein localization provided the first clues. While LGN is localized to the apical side of dividing wild type epidermal cells, it is randomized in p63 null embryos. A similar disruption of the apical mInsc/LGN/NuMA complex is also observed in cells lacking an intact basement membrane [5]. In p63 null embryos basement membrane integrity was lost as judged by staining with β1-integrin and laminin, suggesting that the effect of p63 could be mediated through disruption of basement membrane. This effect of p63 deletion on the basement membrane was consistent with a previous study reporting its role in maintaining cell adhesion and basement membrane integrity [41]. In particular, that study had identified core basement membrane components as targets of p63. The basement membrane defects result in a loss of cell polarity as evidenced by mislocalization of Par3 and aPKCζ and by loss of the activating phosphorylation of aPKCζ. Thus p63 does not appear to play a direct role in controlling the expression of ACD machinery. However, it remains unclear whether p63 has additional targets that promote polarity or ACD in the epidermis.

## Conclusion

The multi-layered structure of adult skin is initiated from a single layer during embryonic development. At the root of this process is the ability of basal progenitor cells to undergo ACD to generate one suprabasal cell that is committed to differentiate and another basal cell that maintains its progenitor status. The apical-basal spindle orientation in cells undergoing ACD is facilitated by adaptor proteins mInsc and LGN that allow the coupling between apical polarity and spindle orientation pathways. Consistent with this, expression and localization of mInsc is tightly regulated in a development and cell-cycle dependent manner [5,9]. However, the regulation of Par3 and mInsc interaction through the cell cycle requires further research. Additional work is also required to determine if cell-cell adhesion structures are also involved in the correct localization of ACD regulators. Previous work has implicated adherens junctions in the cortical localization of LGN and Par3, and in spindle orientation [5,42]. How adherens junction proteins control cell polarity and spindle orientation is poorly understood. A clear understanding of the roles of polarity and adhesion components in regulation of ACD will be useful to understand how the machinery responds to injuries that disrupt intercellular adhesion and structural integrity.

Furthermore, the current study by Poulson and Lechler revealed that the mechanism of ACD is far from simple and involves regulation at multiple levels [9]. The observations upon prolonged expression of mInsc suggested that 1) apical Insc/LGN could be uncoupled from spindle orientation, 2) NuMA maintenance at the apical cortex is a crucial regulatory point for perpendicular spindle orientation during ACD and 3) basal cells have mechanism/s that sense and respond to imbalances in division orientation. The molecular nature of such a "sensor" requires further investigation but one intriguing candidate is tension in the basal layer of cells.

The role and regulation of spindle orientation during ACD is only beginning to be understood in vertebrates. Deregulation of spindle orientation not only generates developmental defects in epidermal and neuronal cells, but has also been implicated in tumorigenesis in the human gut epithelium, further underscoring its importance [43]. Thorough understanding of the ACD machinery in diverse cell types will be important to fully appreciate their role in normal development and during pathology.

## Competing interests

The authors declare that they have no competing interests.

## Authors' contributions

SR and TL wrote and approved the manuscript.
